# Germline Counterparts of Oncogenic Mutations: Who Gives a JAK?

**DOI:** 10.18632/oncotarget.1105

**Published:** 2013-06-23

**Authors:** Adam J. Mead, Stefan N. Constantinescu, Sten Eirik W. Jacobsen

**Affiliations:** Haematopoietic Stem Cell Biology Laboratory, WIMM, University of Oxford, Oxford, UK; Ludwig Institute for Cancer Research and Université catholique de Louvain, de Duve Institute, Brussels; Haematopoietic Stem Cell Biology Laboratory, WIMM, University of Oxford, Oxford, UK

We have recently described a family with hereditary thrombocytosis caused by an inherited JAK2V617I mutation, the first report of a germline activating JAK2 mutation (Mead et al, *New England Journal of Medicine*,2012;366:967-9). Aside from the implications for diagnostic algorithms in the myeloproliferative neoplasms (MPNs), study of germline JAK2 mutations has the potential to contribute to understanding of the biology of MPNs. More broadly, such germline counterparts of oncogenic mutations offer valuable opportunities to study such mutations in isolation, rather than in the context of clonally complex malignant diseases.

With the advent of next generation sequencing, there is an enormous wealth of genetic information arising from human cancers with opportunities to translate these data to improve the care of patients through refined patient stratification, personalised treatment strategies and improved understanding of disease biology. However, with these opportunities also come considerable challenges, not in the least due to the multiple underlying layers of complexity within each malignancy, with various collaborating somatic mutations occurring in different subclones derived from distinct target cells within the respective cellular hierarchy. Furthermore, these subclones can also be differentially selected for during therapy and at disease progression. A number of experimental approaches can be used to understand the impact of individual mutations within the context of such clonally complex disease:

- Correlative studies in large patient populations
- Laboratory models of human mutations in cell lines or animals
- Clonal studies of highly purified human cell populations from tumours, including single cell analyses
- Studies of germline human mutations

The myeloproliferative neoplasms (MPNs) offer a tractable model disease to apply these approaches to help unravel this complexity. Since their first description in 2005, somatic JAK2 mutations are now well established as a key pathogenic event in MPNs, as supported by multiple animal models and cell line studies, as well as clonal analysis of human samples. These approaches, whilst informative, are also associated with limitations. Thus, whilst it is unequivocally established that somatic JAK2 mutations have a key role in driving the MPN pathogenesis, some important questions remain. For example, it remains unclear whether JAK2 mutations in humans are sufficient, as an isolated genetic event, to induce a fully penetrant myeloproliferative phenotype. Indeed, the JAK2V617F mutation is known to occur rarely in patients without any overt haematopoietic phenotype. Further, JAK2 mutations are a secondary event in certain cases of MPN, where they are detectable in only a small subclone of cells. Together, these findings have hampered understanding of the impact of JAK2 mutations on the haematopoietic hierarchy, with studies in both mouse models and humans showing diverse stem/progenitor cell phenotypes, which might be attributable to the JAK2 mutation, or alternatively due to other collaborating genetic events.

We recently addressed some of these questions through a more detailed genetic and phenotypic analysis of patients carrying a germline JAK2V617I mutation (Mead et al, *Blood*,2013;121:4156-65). Using a combination of clonality studies, single nucleotide polymorphism array, targeted mutation screening and exome sequencing we demonstrated that the germline JAK2V617I mutation is likely to be the sole genetic event in these patients, in keeping with the high penetrance and young age of onset of the thrombocytosis phenotype. Analysis of the haematopoietic hierarchy showed a probable expansion of the haematopoietic stem cell pool in cases carrying the JAK2V617I mutation, supported by xenograft studies. This is consistent with the notion that JAK2 mutations might provide a clonal advantage to propagating HSCs during MPN pathogenesis. However, this also highlights a clear limitation of the study of germline mutations, which per definition do not arise as a somatic mutation in a single cell, which must then undergo clonal expansion.

Besides the prevalent JAK2V617F mutation, among V617L/M/I/W, JAK2V617I is the only activating V617 mutation seen in MPN patients, although much less frequent (Dusa et al JBC, 2008;283, 12941-12948). Importantly, we demonstrated clear signaling differences between V617F and V617I mutants, with JAK2V617I resulting in constitutive signaling which was much weaker than seen with JAK2V617F, although both mutations caused similar levels of cytokine hyper-responsiveness. This highlights the potential to compare and contrast the signaling consequences of different germline mutations and correlate this with clinical phenotypes. In this regard, it is noteworthy that multiple diverse germline JAK2 mutations have now been reported in abstract form, with even more kindreds likely to follow. Correlation of mutation-specific clinical phenotypes with distinct signaling events has obvious potential to translate through to patient benefit.

More broadly, we believe that the identification and study of germline mutations predisposing to a malignant phenotype in blood diseases, and beyond, can make a significant contribution to understanding the biology of these mutations; for example, these studies will help to clarify whether and how such germline mutations require clonal evolution through acquisition of additional mutations before a truly “neoplastic” phenotype emerges. In haematopoietic diseases alone, germline counterparts of somatic mutations have been reported to occur in signaling pathway genes e.g. *Mpl* and key transcription factors e.g. *Gata2*, *Runx1* and *Cebpa*. There is a strong argument to develop robust registries and sample banks of these patients, not in the least to provide clinical information as to the likely implication for carriers of these rare mutations, but also to facilitate the above biological studies.

